# Three Improvements in Reduction and Computation of Elliptic Integrals

**DOI:** 10.6028/jres.107.034

**Published:** 2002-10-01

**Authors:** B. C. Carlson

**Affiliations:** Ames Laboratory and Department of Mathematics, Iowa State University, Ames, IA 50011-3020

**Keywords:** computational algorithm, elementary symmetric function, elliptic integral, hyperelliptic integral, hypergeometric *R*-function, recurrence relations, series expansion

## Abstract

Three improvements in reduction and computation of elliptic integrals are made. 1. Reduction formulas, used to express many elliptic integrals in terms of a few standard integrals, are simplified by modifying the definition of intermediate “basic integrals.” 2. A faster than quadratically convergent series is given for numerical computation of the complete symmetric elliptic integral of the third kind. 3. A series expansion of an elliptic or hyperelliptic integral in elementary symmetric functions is given, illustrated with numerical coefficients for terms through degree seven for the symmetric elliptic integral of the first kind. Its usefulness for elliptic integrals, in particular, is important.

## Foreword

Elliptic integrals have many applications, for example in mathematics and physics:

- arclengths of plane curves (ellipse, hyperbola, Bernoulli’s lemniscate)
- surface area of an ellipsoid in 3-dimensional space
- electric and magnetic fields associated with ellipsoids
- periodicity of anharmonic oscillators
- mutual inductance of coaxial circles
- age of the universe in a Friedmann model

These applications are mentioned in the chapter on elliptic integrals, written by B. C. Carlson, that will appear in the *NIST Digital Library of Mathematical Functions*.

The DLMF is scheduled to begin service in 2003 from a NIST Web site. A hardcover book will be published also. These resources will provide a complete guide to the higher mathematical functions for use by experienced scientific professionals. The book will provide mathematical formulas, references to proofs, references to extensions and generalizations, graphs, brief descriptions of computational methods, a survey of useful published tables, and sample applications. The Web site will include, in addition, interactive visualizations of 3-dimensional surfaces, a mathematics-aware search engine, a downloading capability for equations, live links to Web sites that provide mathematical software, and a limited facility for generating tables on demand.

The DLMF is modeled after the *Handbook of Mathematical Functions*, published in 1964 by the National Bureau of Standards with M. Abramowitz and I. A. Stegun as editors. This handbook has been enormously successful: it has sold more than 500,000 copies, its sales remain high, and it is very frequently cited in journal articles in physics and many other fields. But new properties of the higher functions have been developed, and new functions have risen in importance in applications, since the publication of the Abramowitz and Stegun handbook. More than half of the old handbook was devoted to tables, now made obsolete by the revolutionary improvements since 1964 in computers and software. The need for a modern reference is being filled by more than 30 expert authors who are working under contract to NIST and supervised by four NIST editors. The writing is being edited carefully to assure consistent style and level of content.

Elliptic integrals have long been associated with the name of Legendre. Legendre’s *incomplete elliptic integrals* are

$$\begin{array}{l} F\left(\phi ,\;k\right)=\int_{0}^{\phi }\frac{d\theta }{\sqrt{1-k^{2}{\text{sin}}^{2}\theta }},\\ E\left(\phi ,\;k\right)=\int_{0}^{\phi }\sqrt{1-k^{2}\;{\text{sin}}^{2}\;\theta }\;d\theta , \end{array}$$

and

$$\prod \left(\phi ,\alpha^{2},k\right)=\int_{0}^{\phi }\frac{d\theta }{\sqrt{1-k^{2}{\text{sin}}^{2}\theta }\left(1-\alpha^{2}{\text{sin}}^{2}\theta \right)}.$$

The complete forms of these integrals are obtained by setting *ϕ* = π/2.

Over a period of more than 35 years, Carlson has published a series of papers that provide valuable new mathematical and computational foundations for the subject in terms of the *symmetric elliptic integrals*

$$\begin{array}{c} R_{F}\left(x,y,z\right)=\frac{1}{2}\int_{0}^{\infty }\frac{dt}{s\left(t\right)},\\ R_{D}\left(x,y,z\right)=\frac{3}{2}\int_{0}^{\infty }\frac{dt}{s\left(t\right)\left(t+z\right)},\\ R_{J}\left(x,y,z,p\right)=\frac{3}{2}\int_{0}^{\infty }\frac{dt}{s\left(t\right)\left(t+p\right)}, \end{array}$$

where

$$s\left(t\right)=\sqrt{t+x}\sqrt{t+y}\sqrt{t+z}.$$

The complete forms are obtained by setting *x* = 0. In comparison with Legendre’s integrals, Carlson’s integrals simplify the reduction of general elliptic integrals to standard forms and open the way to efficient computations by application of a duplication theorem.

One of the purposes of the DLMF project is to stimulate research into the theory, computation and application of the higher mathematical functions. The paper which follows is an example. It is a further development of material that appears in Carlson’s DLMF chapter on elliptic integrals.

Daniel W. Lozier

NIST Mathematical and Computational Sciences Division

## 1. Simplified Formulas for Reducing Elliptic Integrals

A large class of elliptic integrals can be written in the form

$$I\left(m\right)=\int_{y}^{x}\prod_{i=1}^{h}{\left(a_{i}+b_{i}t\right)}^{-1/2}\prod_{j=1}^{n}{\left(a_{j}+b_{j}t\right)}^{m_{j}}dt,$$ (1.1)

where **m** = (*m*_1_, …, *m_n_*) is an *n*-tuple of integers (positive, negative or zero), *x* > *y*, *h* = 3 or 4, *n* ≥ *h*, and the different linear factors are not proportional. The *a*’s and *b*’s may be complex (with the *b*’s not equal to zero), but the integral is assumed to be well defined, possibly as a Cauchy principal value. In particular the line segment with endpoints *a_i_* + *b_i_x* and *a_i_* + *b_i_y* is assumed to lie in the cut plane ℂ\(−∞,0)for 1 ≤ *i* ≤ *h*.

We write $m=\sum_{j=1}^{n}m_{j}e_{j}$, where **e***_j_* is an *n*-tuple with 1 in the *j* th position and 0’s elsewhere, and we define **0** = (0, …, 0). Reference [1] gives a general method of reducing *I* (**m**) to a linear combination of “basic integrals,” defined as *I* (**0**), *I* (−**e***_j_*), 1 ≤ *j* ≤ *n*, and (if *h* = 4) *I* (**e***_k_*), 1 ≤ *k* ≤ 4. A simple example of such a reduction is

$$\begin{array}{c} b_{i}I\left(e_{j}-e_{i}\right)=d_{ji}I\left(-e_{i}\right)+b_{j}I\left(0\right),\\ i,j\in \left\{1,\ldots ,n\right\}, \end{array}$$ (1.2)

where *d_ji_* = *a_j_b_i_* − *a_i_b_j_*. This equation reduces all 36+72 integrals in Ref. [2], Eqs. (3.142) and (3.168) and also the 18 integrals in Ref. [2], Eq. (3.159) after taking *x*^2^ as a new variable of integration. The basic integrals are expressed in terms of symmetric standard integrals *R_F_*, *R_D_*, and *R_J_* in Ref. [1], Sec. 4.

The general method first reduces *I* (**m**) by Ref. [1], Eq. (2.19) to integrals in which **m** has at most one nonzero component and then uses two recurrence relations Ref. [1] Eqs. (3.5) and (3.11) for further reduction to basic integrals. For example, the simplest recurrence relation is Ref. [1], Eq. (3.11):

$$b_{i}^{q}I\left(qe_{j}\right)=\sum_{r=0}^{q}\left(\begin{array}{l} q\\ r \end{array}\right)b_{j}^{r}d_{jt}^{q-r}I\left(re_{i}\right),\;q\in \mathbb{N}.$$ (1.3)

The *b*’s appear also in the other two formulas and therefore in all reduction formulas, sometimes in great profusion if **m** is considerably more complicated than in Eq. (1.2).

It has been found that the *b*’s disappear from all three formulas, and therefore from all reduction formulas, if we define

$$\begin{array}{l} \hat{I}\left(m\right)=I\left(m/B\right),\hat{A}\left(m\right)=A\left(m\right)/B,\\ B=\prod_{j=1}^{n}b_{j}^{m_{j}},\;r_{ij}=\frac{d_{ij}}{b_{i}b_{j}}=\frac{a_{i}}{b_{i}}-\frac{a_{j}}{b_{j}}. \end{array}$$ (1.4)

Here *A* (**m**) is the algebraic function

$$A\left(m\right)=f_{m}\left(x\right)-f_{m}\left(y\right),$$ (1.5)

where *f***_m_**(*t*) is the integrand of Eq. (1.1). Note that ***Î***(**0**) = ***I***(**0**).

For example, Eq. (1.2) becomes

$$\hat{I}\left(e_{j}-e_{i}\right)=r_{ji}\hat{I}\left(-e_{i}\right)+I\left(0\right),$$ (1.6)

and Eq. (1.3) becomes

$$\hat{I}\left(qe_{j}\right)=\sum_{r=0}^{q}\left(\begin{array}{l} q\\ r \end{array}\right)r_{ji}^{q-r}\hat{I}\left(re_{i}\right),\;q\in \mathbb{N}.$$ (1.7)

The other recurrence relation, Ref. [1], Eq. (3.5), becomes

$$\begin{array}{l} \sum_{r=0}^{h}\left(2q+r\right)E_{h-r}\left(r_{1j},\ldots ,r_{hj}\right)\hat{I}\left(\left(q-1+r\right)e_{j}\right)\\ =2\hat{A}\left(qe_{j}+\sum_{i=1}^{h}e_{i}\right),\;q\in \mathbb{Z},\;1\leq j\leq n \end{array}$$ (1.8)

where *E_h_*_−_*_r_* is the elementary symmetric function defined by

$$\prod_{i=1}^{h}\left(1+tr_{ij}\right)=\sum_{k=0}^{h}t^{k}E_{k}\left(r_{1j}\ldots ,r_{hj}\right),$$ (1.9)

whence *E_h_* = 0 if 1 ≤ *j* ≤ *h* because *r_jj_* = 0.

The remaining formula, Ref. [1], Eq. (2.19), becomes

$$\hat{I}\left(m\right)=\sum_{q=0}^{M}{\hat{C}}_{M-q}\left(k\right)\hat{I}\left(qe_{k}\right)+\sum_{i=1}^{n}\Delta_{i}\sum_{q=1}^{-m_{i}}{\hat{C}}_{m_{i}+q}\left(i\right)\hat{I}\left(-qe_{i}\right),$$ (1.10)

where $M=\sum_{j=1}^{n}m_{j}$ and each sum is empty if its upper limit is less than its lower limit. The first term on the right is independent of *k*, which is usually best chosen so that 1 ≤ *k* ≤ *h*. The coefficients are defined by

$$\begin{array}{l} \Delta_{i}=\prod_{\begin{array}{l} j=1\\ j\neq i \end{array}}^{n}r_{ji}^{m_{j}},\;{\hat{C}}_{0}\left(i\right)=1,\;{\hat{C}}_{\pm s}\left(i\right)=\sum \frac{\eta_{\pm 1}^{\alpha_{1}}\left(i\right)\cdots \eta_{\pm s}^{\alpha_{s}}\left(i\right)}{\alpha_{1}!\cdots \alpha_{s}!},\\ \eta_{\pm p}\left(i\right)=\frac{-1}{p}\sum_{\begin{array}{l} j=1\\ j\neq i \end{array}}^{n}m_{j}r_{ij}^{\pm p},\;p\geq 1, \end{array}$$ (1.11)

where upper (lower) signs go together and the first sum extends over all nonnegative integers *α*_1_, …, *α_s_* such that *α*_1_ + 2 *α*_2_ + … + *sα_s_* = *s*. A recurrence relation for the coefficients is

$$s{\hat{C}}_{\pm s}\left(i\right)=\sum_{p=1}^{s}p\eta_{\pm p}\left(i\right){\hat{C}}_{\pm \left(s-p\right)}\left(i\right),\;s\geq 1.$$ (1.12)

## 2. Algorithms for Complete Elliptic Integrals of the Third Kind

Complications formerly encountered in numerical computation of Legendre’s complete elliptic integral of the third kind were avoided by defining and tabulating Heuman’s Lambda function (for circular cases) and a modification of Jacobi’s Zeta function (for hyperbolic cases). For example, the method of Ref. [3] was later superseded by Bartky’s transformation and its application by Bulirsch [4] to his complete integral *cel* (*k_c_*, *p*, *a*, *b*). Bartky’s transformation for the complete case of the symmetric integral of the third kind, obtained from Ref. [5], Eq. (4.14) with the help of

$$\begin{array}{l} \left(3\pi /4\right)R_{L}\left(y,z,p\right)=3R_{F}\left(0,y,z\right)-pR_{J}\left(0,y,z,p\right),\\ \;\left(\pi /2\right)R_{K}\left(y,z\right)=R_{F}\left(0,y,z\right), \end{array}$$ (2.1)

can be written as

$$\begin{array}{l} R_{J}\left(0,g_{n}^{2},a_{n}^{2},p_{n}^{2}\right)=s_{n}R_{J}\left(0,g_{n+1}^{2},a_{n+1}^{2},p_{n+1}^{2}\right)\\ +\left(3/2p_{n}^{2}\right)R_{F}\left(0,g_{n+1}^{2},a_{n+1}^{2}\right), \end{array}$$ (2.2)

where *a_n_*, *g_n_*, *p_n_* are positive, $S_{n}=\left(p_{n}^{4}-a_{n}^{2}g_{n}^{2}\right)/8p_{n}^{4}$ and

$$\begin{array}{c} a_{n+1}=\frac{a_{n}+g_{n}}{2},\;g_{n+1}=\sqrt{a_{n}g_{n}},\;p_{n+1}=\frac{p_{n}^{2}+a_{n}g_{n}}{2p_{n}},\\ n\in \mathbb{N}. \end{array}$$ (2.3)

As *n* → ∞, *a_n_* and *g_n_* converge quadratically to Gauss’s arithmetic-geometric mean, *M* (*a*_0_, *g*_0_), and

$$R_{F}\left(0,g_{n}^{2},a_{n}^{2}\right)=\pi /2M\left(a_{0},g_{0}\right),\;n\in \mathbb{N},$$ (2.4)

by Ref. [6], Eqs. (6.10–8) and Eq. (2.1). It follows from (2.3) that

$$p_{n+1}-g_{n+1}={\left(p_{n}-g_{n+1}\right)}^{2}/2p_{n}.$$ (2.5)

Since *g_n_*_+1_ converges quadratically to *M*(*a*_0_, *g*_0_), so does *p_n_*, and *s_n_* converges quadratically to 0. Iteration of Eq. (2.2) gives

$$\begin{array}{c} R_{J}\left(0,g_{0}^{2},a_{0}^{2},p_{0}^{2}\right)=\frac{Q_{n}p_{n}^{2}}{p_{0}^{2}}R_{J}\left(0,g_{n}^{2},a_{n}^{2},p_{n}^{2}\right)\\ +\frac{3\pi }{4p_{0}^{2}M\left(a_{0},g_{0}\right)}\sum_{m=0}^{n-1}Q_{m}, \end{array}$$ (2.6)

where

$$Q_{0}=1,\;\frac{Q_{m}}{p_{0}^{2}}=\frac{s_{0}s_{1}\cdots s_{m-1}}{p_{m}^{2}},\;m\geq 1,$$ (2.7)

and therefore

$$\frac{Q_{n+1}}{Q_{n}}=\frac{s_{n}p_{n}^{2}}{p_{n+1}^{2}}=\frac{1}{2}\frac{p_{n}^{2}-a_{n}g_{n}}{p_{n}^{2}+a_{n}g_{n}}.$$ (2.8)

Letting *n* → ∞ in Eq. (2.6), we find, for positive *a*_0_, *g*_0_, *p*_0_,

$$\begin{array}{l} R_{J}\left(0,g_{0}^{2},a_{0}^{2},p_{0}^{2}\right)=\frac{3\pi }{4p_{0}^{2}M\left(a_{0},g_{0}\right)}\sum_{n=0}^{\infty }Q_{n},\\ Q_{0}=1,\;Q_{n+1}=\frac{1}{2}Q_{n}\epsilon_{n}\;\epsilon_{n}=\frac{p_{n}^{2}-a_{n}g_{n}}{p_{n}^{2}+a_{n}g_{n}}, \end{array}$$ (2.9)

where ϵ*_n_* converges to 0 quadratically and *Q_n_* converges to 0 faster than quadratically.

If *p*_0_ = *a*_0_, Eq. (2.9) becomes

$$\begin{array}{l} R_{D}\left(0,g_{0}^{2},a_{0}^{2}\right)=\frac{3\pi }{4a_{0}^{2}M\left(a_{0},g_{0}\right)}\sum_{n=0}^{\infty }Q_{n},\\ Q_{0}=1,\;Q_{n+1}=\frac{1}{2}Q_{n}\epsilon_{n},\;\epsilon_{n}=\frac{a_{n}-g_{n}}{a_{n}+g_{n}}, \end{array}$$ (2.10)

where *R_D_* is a complete integral of the second kind, symmetric in only its first two arguments. If 0 < *a*_0_ ≤ *g*_0_ then −1 < ϵ_0_ ≤ 0, but ϵ*_n_* ≥ 0 and *Q_n_ ≤* 0 for *n* ≥ 1.

If the last variable of *R_J_* is negative, the Cauchy principal value is given by

$$\begin{array}{l} \left(q_{0}^{2}+a_{0}^{2}\right)R_{J}\left(0,g_{0}^{2},a_{0}^{2},-q_{0}^{2}\right)=\left(p_{0}^{2}-a_{0}^{2}\right)R_{J}\left(0,g_{0}^{2},a_{0}^{2},p_{0}^{2}\right)\\ -\frac{3\pi }{2M\left(a_{0},g_{0}\right)},p_{0}^{2}=a_{0}^{2}\left(q_{0}^{2}+g_{0}^{2}\right)/\left(q_{0}^{2}+a_{0}^{2}\right), \end{array}$$ (2.11)

where we have used Eq. (2.4) and chosen $x_{i}=a_{0}^{2}$ in Ref. [7], Eq. (4.6). Substitution of Eq. (2.9) gives

$$\begin{array}{c} R_{J}\left(0,g_{0}^{2},a_{0}^{2},-q_{0}^{2}\right)=\frac{-3\pi }{4M\left(a_{0},g_{0}\right)\left(q_{0}^{2}+a_{0}^{2}\right)}\\ \left(2+\frac{a_{0}^{2}-g_{0}^{2}}{q_{0}^{2}+g_{0}^{2}}\sum_{n=0}^{\infty }Q_{n}\right). \end{array}$$ (2.12)

Equation (2.3) and the second line of Eq. (2.9) still apply, with *p*_0_ given by Eq. (2.11).

For the complete case of Legendre’s third integral,

$$\Pi \left(\alpha^{2},k\right)=\left(\alpha^{2}/3\right)R_{J}\left(0,{k^{\prime }}^{2},1,1-\alpha^{2}\right)+R_{F}\left(0,{k^{\prime }}^{2},1\right),$$ (2.13)

where *k*′^2^ = 1 − *k*^2^, Eq. (2.9) implies

$$\begin{array}{c} \Pi \left(\alpha^{2},k\right)=\frac{\pi }{4M\left(1,k^{\prime }\right)}\left(2+\frac{\alpha^{2}}{1-\alpha^{2}}\sum_{n=0}^{\infty }Q_{n}\right),\\ -\infty <k^{2}<1,\;-\infty <\alpha^{2}<1, \end{array}$$ (2.14)

where Eq. (2.3) applies with *a*_0_ = 1, *g*_0_ = *k*′, and $p_{0}^{2}=1-\alpha^{2}$.

If α^2^ > 1, Eq. (2.12) gives the Cauchy principal value,

$$\begin{array}{c} \Pi \left(\alpha^{2},k\right)=\frac{-\pi k^{2}}{4M\left(1,k^{\prime }\right)\left(\alpha^{2}-k^{2}\right)}\sum_{n=0}^{\infty }Q_{n},\\ -\infty <k^{2}<1,\;1<\alpha^{2}<\infty , \end{array}$$ (2.15)

where Eq. (2.3) and the second line of Eq. (2.9) apply with *a*_0_ = 1, *g*_0_ = *k*′, and $p_{0}^{2}=1-k^{2}/\alpha^{2}$.

## 3. Expansion in Elementary Symmetric Functions

The duplication method of computing the symmetric elliptic integrals *R_F_* and *R_J_* (including their degenerate cases *R_C_* and *R_D_*) consists in iterating their duplication theorems until their variables are nearly equal and then expanding in a series of elementary symmetric functions of the small differences between the variables. In the absence of a duplication theorem the method is useful for a hyperelliptic integral only if the variables are nearly equal. The series is truncated to a polynomial of fixed degree; the higher the degree, the fewer duplications are needed for a desired accuracy of the result but the larger the number of terms to be calculated. No tests have been made to determine an optimal compromise, which would depend in large part on the speed of extracting square roots in the duplication theorem and therefore on the equipment used. In Ref. [8] polynomials of degree five were chosen for simplicity, but later it seemed worthwhile to increase the degree for the comparatively slow computation of *R_J_*, and Ref. [9], Eq. (A.11) gives the terms of degree six and seven for *R_J_*. The change in speed would be significant only if a very large number of computations were performed, since the result of a single computation is returned with no delay apparent to the eye. We shall give here the corresponding terms for *R_F_* and the general form of the infinite series of which the polynomials are truncations.

Any *R*-function *R*_−_*_a_*(*b*_1_, …, *b_k_*; *z*_1_, …, *z_k_*) (see Ref. [6]) in which all the *b*-parameters are positive integral multiples of a single number *β* can be rewritten with repeated variables and all *b*’s equal to *β*. An example is

$$\begin{array}{l} R_{J}\left(x,y,z,p\right)=R_{-3/2}(\frac{1}{2},\frac{1}{2},\frac{1}{2},1;x,y,z,p)\\ \;=R_{-3/2}(\frac{1}{2},\frac{1}{2},\frac{1}{2},\frac{1}{2},\frac{1}{2};x,y,z,p,p), \end{array}$$ (3.1)

and *R_D_* (*x*, *y*, *z*) is the case with *p* = *z*. Therefore we consider

$$\begin{array}{l} \;R_{-a}(\beta ,\ldots ,\beta ;z_{1},\ldots ,z_{n})\\ =\frac{1}{B\left(a,n\beta -a\right)}\int_{0}^{\infty }t^{n\beta -a-1}\prod_{i=1}^{n}{\left(t+z_{i}\right)}^{-\beta }dt\\ =A^{-a}R_{-a}\left(\beta ,\ldots ,\beta ;z_{1}/A,\ldots ,z_{n}/A\right), \end{array}$$ (3.2)

where *B* is the beta function and we have used the homogeneity of *R* to divide the variables by their arithmetic average,

$$A=\frac{1}{n}\sum_{i=1}^{n}z_{i}.$$ (3.3)

The relative difference between *A* and *z_i_* is

$$Z_{i}=\frac{A-z_{i}}{A}=1-\frac{z_{i}}{A},$$ (3.4)

whence

$$\begin{array}{l} R_{-a}(\beta ,\ldots ,\beta ;z_{1},\ldots ,z_{n})\\ =A^{-a}R_{-a}(\beta ,\ldots ,\beta ;1-Z_{1},\ldots ,1-Z_{n}). \end{array}$$ (3.5)

Because the function is symmetric in the *Z*’s, it can be expanded in elementary symmetric functions *E_m_* = *E_m_*(*Z*_1_, …, *Z_n_*) defined by

$$\prod_{i=1}^{n}(1+tZ_{i})=\sum_{r=0}^{n}t^{r}E_{r}.$$ (3.6)

Applying Ref. [10], Eqs. (A.5) and (A.12) to the right side of Eq. (3.5), we find

$$\begin{array}{l} \;R_{-a}(\beta ,\ldots ,\beta ;z_{1},\ldots z_{n})\\ =A^{-a}\sum_{N=0}^{\infty }\frac{{\left(a\right)}_{N}}{{\left(n\beta \right)}_{N}}T_{N}(\beta ,\ldots ,\beta ;Z_{1},\ldots ,Z_{n}), \end{array}$$ (3.7)

$$=A^{-a}\sum_{N=0}^{\infty }\frac{{\left(a\right)}_{N}}{{\left(n\beta \right)}_{N}}\sum {\left(-1\right)}^{N+M}{\left(\beta \right)}_{M}\frac{E_{1}^{m_{1}}\cdots E_{n}^{m_{n}}}{m_{1}!\cdots m_{n}!},$$ (3.8)

where (*a*)*_N_* is Pochhammer’s shifted factorial, $M=\sum_{i}^{n}m_{i}$, and the inner sum (representing *T_N_*) extends over all nonnegative integers *m*_1_, …, *m_n_* such that *m*_1_ + 2*m*_2_ + … + *nm_n_* = *N*. Reference [10], Eq. (A.6) provides the recurrence relation

$$NT_{N}+\sum_{r=2}^{n}{\left(-1\right)}^{r}\left(r\beta +N-r\right)E_{r}T_{N-r}=0,$$ (3.9)

where *T*_0_ = 1 and *T_N_*_−_*_r_* = 0 if *r* > *N*, whence *T*_1_ = 0. The term with *r* = 1 is missing because Eqs. (3.3) and (3.4) imply *E*_1_ = 0, which greatly simplifies *T_N_*.

Let |*Z*| = max*_i_* |*Z_i_*| and *λ* = max{|*a*|, 1}. The series [Eq. (3.7)] converges absolutely if *β* > 0 and |*Z*| < 1, and the truncation error *r_K_* resulting from neglect of terms of degree *N* ≥ *K* can be shown to satisfy

$$\left|r_{K}\right|\leq \frac{{\left(\left|a\right|\right)}_{K}{\left|Z\right|}^{K}}{K!{\left(1-\left|Z\right|\right)}^{\lambda }}.$$ (3.10)

Each application of a duplication theorem decreases by a factor of four the differences between the variables, therefore the difference *A* − *z_i_*, and ultimately |*Z*| as *A* approaches a limit. It is easy to determine whether another duplication is needed before using the truncated series to achieve the desired accuracy.

If *a* = *β* = 1/2 and *n* = 3, the series [Eq. (3.8)] with *E*_1_ = 0 can be rearranged as

$$R_{F}(z_{1},z_{2},z_{3})=A^{-1/2}\sum_{r=0}^{\infty }\sum_{s=0}^{\infty }\frac{{\left(-1\right)}^{r}{\left(\frac{1}{2}\right)}_{r+s}}{4r+6s+1}\frac{E_{2}^{r}E_{3}^{s}}{r!s!}.$$ (3.11)

This double series is convenient for obtaining numerical coefficients but requires |*E*_2_| + |*E*_3_| < 1 for absolute convergence. Keeping together all terms of the same degree in the *Z*’s, as in Eq. (3.8), we find

$$R_{F}(z_{1},z_{2},z_{3})=A^{-1/2}(1-\frac{1}{10}E_{2}+\frac{1}{14}E_{3}+\frac{1}{24}E_{2}^{2}-\frac{3}{44}E_{2}E_{3}-\frac{5}{208}E_{2}^{3}+\frac{3}{104}E_{2}^{2}E_{3}+r_{8}),$$ (3.12)

where

$$\begin{array}{c} \left|r_{8}\right|<\frac{0.2{\left|Z\right|}^{8}}{1-\left|Z\right|},\;\left|Z\right|=\underset{i}{\max}\left|\frac{A-z_{i}}{A}\right|<1,\\ A=\left(z_{1}+z_{2}+z_{3}\right)/3. \end{array}$$ (3.13)

One more duplication would (ultimately) have decreased |*r*_8_| by a factor of 4^8^ = 65 536.

For convenient reference we restate the corresponding result for *R_J_* and *R_D_* [see Eq. (3.1) and the discussions preceding it]:

$$\begin{array}{l} \;R_{-3/2}\left(\frac{1}{2},\ldots ,\frac{1}{2};z_{1},\ldots ,z_{5}\right)\\ \;=A^{-3/2}(1-\frac{3}{14}E_{2}+\frac{1}{6}E_{3}+\frac{9}{88}E_{2}^{2}-\frac{3}{22}E_{4}\\ -\frac{9}{52}E_{2}E_{3}+\frac{3}{26}E_{5}-\frac{1}{16}E_{2}^{3}+\frac{3}{40}E_{3}^{2}+\frac{3}{20}E_{2}E_{4}\\ +\frac{45}{272}E_{2}^{2}E_{3}-\frac{9}{68}E_{3}E_{4}-\frac{9}{68}E_{2}E_{5}+r_{8}), \end{array}$$ (3.14)

where

$$\begin{array}{c} \left|r_{8}\right|<\frac{3.4{\left|Z\right|}^{8}}{{\left(1-\left|Z\right|\right)}^{3/2}},\;\left|Z\right|=\underset{i}{\max}\left|\frac{A-z_{i}}{A}\right|<1,\\ A=\left(z_{1}+\ldots +z_{5}\right)/5. \end{array}$$ (3.15)

The duplication theorems for these two functions are more complicated than that for *R_F_*; see Ref. [10] and Ref. [9], Appendix.
